# The Winding Road of Cardiac Regeneration—Stem Cell Omics in the Spotlight

**DOI:** 10.3390/cells7120255

**Published:** 2018-12-07

**Authors:** Miruna Mihaela Micheu, Alina Ioana Scarlatescu, Alexandru Scafa-Udriste, Maria Dorobantu

**Affiliations:** 1 Department of Cardiology, Clinical Emergency Hospital of Bucharest, Floreasca Street 8, 014461 Bucharest, Romania; alina.scarlatescu@gmail.com (A.I.S.); alexscafa@yahoo.com (A.S.-U.); maria.dorobantu@gmail.com (M.D.); 2 Department 4-Cardiothoracic Pathology, University of Medicine and Pharmacy Carol Davila, Eroii Sanitari Bvd. 8, 050474 Bucharest, Romania

**Keywords:** stem cells, cardiac regeneration, omics data, cardioprotection, precision medicine

## Abstract

Despite significant progress in treating ischemic cardiac disease and succeeding heart failure, there is still an unmet need to develop effective therapeutic strategies given the persistent high-mortality rate. Advances in stem cell biology hold great promise for regenerative medicine, particularly for cardiac regeneration. Various cell types have been used both in preclinical and clinical studies to repair the injured heart, either directly or indirectly. Transplanted cells may act in an autocrine and/or paracrine manner to improve the myocyte survival and migration of remote and/or resident stem cells to the site of injury. Still, the molecular mechanisms regulating cardiac protection and repair are poorly understood. Stem cell fate is directed by multifaceted interactions between genetic, epigenetic, transcriptional, and post-transcriptional mechanisms. Decoding stem cells’ “panomic” data would provide a comprehensive picture of the underlying mechanisms, resulting in patient-tailored therapy. This review offers a critical analysis of omics data in relation to stem cell survival and differentiation. Additionally, the emerging role of stem cell-derived exosomes as “cell-free” therapy is debated. Last but not least, we discuss the challenges to retrieve and analyze the huge amount of publicly available omics data.

## 1. Introduction

Morbidity and mortality caused by ischemic heart disease (IHD) and subsequent heart failure (HF) are still high, despite modern treatments. Standard-of-care therapy improves the outcome of patients, but it does not completely block myocytes loss or adverse cardiac remodeling. The need for effective therapeutic options has driven the quest to develop alternative approaches addressing the critical issue of cell loss. Stem cell-based therapy (SCT) aims to restore cardiac function by delivering exogenous cells, which will eventually generate both contractile cells and blood vessels. In addition, transplanted stem cells (SCs) are known to secrete a large array of molecular mediators, including soluble cytokines and growth factors, thereby enhancing myocyte survival and enabling the migration of remote and/or resident cardiac SCs to the site of injury.

**Figure 1:**
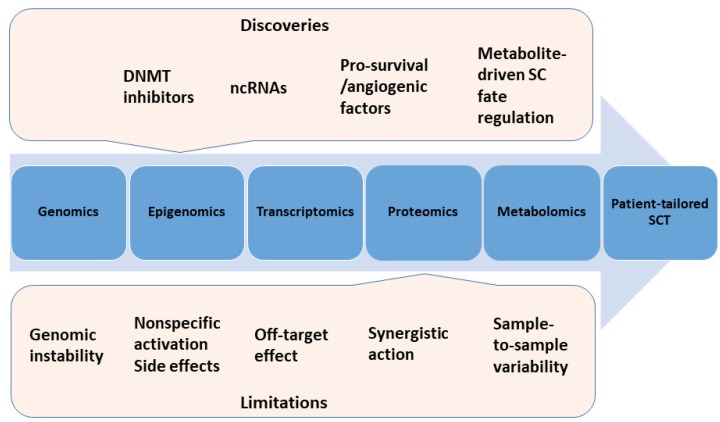
Integrating panomic data in stem cell therapy. Discoveries and limitations have been identified for each category of omic data. Findings that stem cell (SC) fate can be regulated by various factors (such as DNMT inhibitors, ncRNAs, pro-survival or angiogenic factors, and metabolites) provided useful tools to improve cardiac regeneration and achieve patient-tailored therapy. Conversely, there are shortcomings of their use into clinics. Cultured SCs are prone to genomic alterations that affect their differentiation potential and tumorigenicity. The use of DNMT inhibitors is limited by nonspecific transcriptional activation and side effects. Also, ncRNAs as therapeutic agents/targets are hindered by off-target effect due to their ability to regulate genetic networks and not a single pathway. Priming SCs with pro-survival or angiogenic factors and genetic engineering of SCs to overexpress beneficial signals require synergistic action for a significant effect. The use of metabolites to direct SC fate is subjected to sample-to-sample variability in culture condition that hampers the reproducibility of cell culture and differentiation. DNMT = DNA methyltransferase; ncRNAs = non-coding RNAs; SCT = stem cell therapy.

Various types of stem/progenitor cells, manufacturing methods and delivery routes tested in preclinical and clinical settings have been extensively discussed since the inception of the “regenerative era” [1,2,3,4]. Furthermore, “cell-free” therapies comprising the delivery of SCs paracrine factors and/or stem cell-derived extracellular vesicles were also under investigation. Major breakthroughs have been accomplished since the first in-human bone marrow SC transplantation performed in 2001 in IHD [5], but drawbacks and limitations have also been identified [3]. Clinical trials and meta-analyses have revealed a high heterogeneity both in terms of study design and results, raising key issues which are yet to be explored and answered. For example, poor engraftment and survival of the transplanted cells within the ischemic myocardium remains an important shortcoming that impedes long-term cardiac recovery. Prior studies have provided valuable insights in terms of molecular mechanisms and factors that govern these fundamental cell processes. As a result, a number of strategies to overcome the low cell survival rates have been tested, such as priming with pro-survival molecules, preconditioning with hypoxia, and the use of genetic engineering to overexpress antideath or adhesion signals. Hence, a better understanding of the molecular mechanisms of SC-mediated protection and cardiac regeneration is critically needed in order to achieve efficient and safe SCT. In-depth exploration of stem cells’ “panomic” data (i.e., integration of genomics, epigenomics, transcriptomics, proteomics, and metabolomics information) would provide valuable insights into SC biology, eventually achieving the goal of patient-tailored therapy (Figure 1).

## 2. Genomics

While the first trials assessing SCT in IHD employed minimally manipulated heterogeneous cell populations (i.e., bone marrow mononuclear cells), the later trials tested more specific cell subpopulations, or even different cell types (such as mesenchymal stem cells and cardiac stem cells, respectively). One of the downsides of using such cells is the necessity of ex vivo expansion by serial cell culture and passages in order to reach the effective cell dosage. Due to strong selection pressures, long-term cultured SCs are prone to genomic alterations such as point mutations, copy number variations (CNVs) or even large chromosomal aberrations. In time, the aforementioned anomalies are acquired in a large fraction of the cultured cells, tampering their behavior in terms of differentiation capacity and tumorigenicity [6]. The most common genomic abnormalities in cultured human are summarized in Table 1.

From a chronological perspective, the first human SCs analyzed were embryonic stem cells (ESCs), followed by induced pluripotent stem cells (iPSCs). Starting in the early 2000s, a plethora of articles reported a variety of culture-acquired genomic abnormalities, which have been comprehensively discussed in recent reviews [6,7]. The most frequently chromosomal aberration identified in both cell types was trisomy 12, but trisomy 8 and X have also been observed [8]. Of note, these anomalies have functional implications. Enrichment in cell cycle-related and pluripotency-associated genes (such as NANOG and GDF3), due to amplification of chromosome 12, results in selective growth advantage and takeover of cultures by abnormal cells [9]. Single nucleotide variants (SNVs) and CNVs have also been observed in cultured ESCs and iPSCs, 20q11.21 being reported as the most prevalent CNV hotspot. Cells with gain in this region show high-level expression of pluripotency and anti-apoptosis genes harbored in this region, such as DNA methyltransferase 3B, inhibitor of DNA binding, and BCL2-like1 [7,19].

Beside pluripotent SCs, multipotent and progenitor SCs that are expanded in culture are also susceptible to acquiring genomic anomalies. Mesenchymal stem cells (MSCs) are one of the most commonly exploited cell types for treating a variety of medical conditions, so their genome integrity in culture settings has been widely assessed. Existing data suggest that genome stability of human MSCs is preserved in the early stages of culture, but is affected after extended culture.

Although early studies stated that human MSCs retained chromosomal stability following long-term culture, later studies have shown that MSCs are prone to acquiring large chromosomal aberrations, outgrowing the normal cell population within 7 passages. Ben-David and colleagues examined the genetic integrity of 135 human MSC samples from different sources (such as bone marrow, adipose tissue and umbilical cord), reporting a frequency of aberrations of ~4% [15]. Their conclusions are particularly important, since the number of passages of culture-expanded human MSCs that are used in clinical trials is around 5 to 13.

The rate and level of genetic alterations in bone marrow MSCs along serial culture passages were studied by Cai and colleagues by using whole-genome sequencing. There were no substantial alterations in CNV and only low levels of SNVs (0.01%–1%) until the passage 8, but their frequency significantly increased with the number of passages (up to 10% in passage 8 and 17%–36% in passage 13) [20]. These data were confirmed by subsequent work assessing cultured peripheral blood-derived MSCs from passage 1 to 9 [21]. A dramatic growth in SNVs occurrence was detected in later passages, with a frequency of over 70% after passage 7. In addition, the indel incidence displayed a similar pattern, with a steep increase after passage 7, allowing the authors to advocate that similar mutational forces steered the addition of both SNVs and indels. As for CNVs, in line with previous evidence [22], MSCs were proved to basically lack copy number alterations in early passages [21].

Specific attention should be paid to culturing conditions, since they may influence both the type and the prevalence of the acquired aberrations. Oxygen concentration is a key determinant of SCs’ fate, but conflicting results have been reported by studies addressing the effect of hypoxic versus normoxic conditions. In cultured MSCs, hypoxic preconditioning has been revealed to diminish or prevent chromosomal aberrations, but also to enhance structural instability and aneuploidy even at early passages [18].

Although cardiac stem cells (CSCs) are considered to be particularly promising for myocardial regeneration, little data is publicly available about their genomic integrity in ex vivo culture. The evidence is provided by the investigators of the CADUCEUS trial (ClinicalTrials.gov. Identifier NCT00893360), who identified chromosome aberrations (such as trisomy 8 and Y chromosome loss) in about one-third of cells resulting from CSCs grown under normoxic conditions (20% O_2_) [16]. When expanded under physiological low-oxygen conditions (5% O_2_), the frequency of chromosomal abnormalities was significantly reduced [17].

Other types of adult SCs that were analyzed were adipose-derived SCs (ADSCs) and CD34^+^ SCs, respectively. The anomalies (i.e., aneuploidies) were reported starting with passage 2 in the case of ADSCs, while for CD34^+^ cells the karyotype abnormalities mainly appeared by day 14 [18].

So, when dealing with cultured SCs in the clinical arena, the genomic instability is a phenomenon that should not be overlooked, but must be routinely evaluated.

## 3. Epigenomics

Epigenetics denotes changes in gene expression regulation, but not due to alterations in the DNA sequence. These changes are usually a consequence of gene–environment interactions leading to amplified/diminished expression—or even silencing—of specific genes. Although ESCs are not used into clinics as such, but as ESC-derived differentiated cells (ClinicalTrials.gov NCT02057900) [23], they have been extensively exploited as a model to decipher the key factors and mechanisms underlying cell fate decisions [24]. Decoding ESCs epigenome marks resulted in improved strategies for cellular reprogramming and differentiation, which could eventually be translated into clinical settings. Reprogramming of adult somatic cells to a pluripotent state is a typical example of epigenetic modifications, uncovering new approaches to heart regeneration. Since the regenerative potential of adult SCs is often impaired due to modifiers such as age and associated cardiovascular risk factors and comorbidities, there is an unmet need to obtain patient-derived cells with superior clinical performance. Obtained iPSCs can differentiate into various types of cells, including cardiomyocytes and vascular cells, and thus providing unlimited supplies of autologous cells lacking the risk of immune rejection. 

There are three main types of epigenetic modifications: DNA methylation, histone modification, and microRNAs-mediated gene regulation [25]. Since microRNAs fit in both EPIGENOMICS and TRANSCRIPTOMICS; they will be discussed in a later chapter.

### 3.1. DNA Methylation

DNA methylation is one of the key mechanisms that regulates gene expression, being indispensable for both cell differentiation and reprogramming. DNA methyltransferases (DNMTs) are a family of enzymes responsible for maintaining and/or introducing DNA methylation marks. DNA methylation patterns are maintained during cell division by DNA methyltransferase 1 (DNMT1), while de novo DNA methylation is mediated by DNMT3A and DNMT3B. Removal of DNA methylation involves oxidation of 5-methyl-cytosine; the key enzymes for this initial step are the recently discovered ten-eleven translocation enzymes (TET1-3) [26].

Methylation in the promoter of genes causes chromatin condensation and consequently gene silencing [25]. In mammals, it usually consists of methylation of CpG islands, but non-CpG methylation has also been reported, primarily in pluripotent cells. Different populations of SCs have different signatures in terms of DNA methylation. Comprehensive studies of human pluripotent and somatic cell methylomes have provided substantial data related to the epigenetic profile of various cell lines. In this respect, Meissner and colleagues analyzed a panel of 20 ESC lines, 12 iPSC lines and 10 somatic cells [27,28]. Whereas both ESCs and iPSCs exhibited high levels of non-CpG methylation, as opposed to somatic cells, there were also differences in DNA methylation patterns within those specific pluripotent cell lines. When comparing ESCs and iPSCs in terms of DNA methylation and gene expression levels, they proved to form two partially overlapping clusters with variability among both ESCs and iPSCs lines. This finding emphasizes the need to identify the most suitable cell line specifically for each application [27].

Various DNA-demethylating agents have been tested, aiming to prompt the expression of hypermethylated silenced genes. One of the most utilized DNMT inhibitors is 5-azacytidine (5-aza), but its effect on SCs remains contradictory. Although a number of in vitro studies have shown that treatment with 5-aza promoted differentiation of adult MSCs into cardiac muscle-like cells and increased the expression of cardiac-specific proteins [29,30,31,32], others have failed to induce a cardiomyogenic phenotype in treated cells [33,34]. Bearing in mind the mechanisms of action, 5-aza is more likely to induce a general transcriptional activation, instead of differentiation into a specific cell type. Thus, 5-aza-treated rat adult MSCs displayed an increased expression of muscle-specific genes (GATA-4, myoD, desmin, and α-actinin), but also activation of endothelial and neural specific genes [35].

However, beside inconsistency in promoting the differentiation of treated SCs to cardiomyocytes, 5-aza also has side effects (e.g., myeloid suppression) which limit its use in clinical settings. So, other inhibitors have been proposed. Zebularine, with similar effects of promoting differentiation of MSCs into cardiomyocytes with increased expression of cardiac-specific genes, has less toxic effects, making it a more suitable candidate for future studies [36,37].

The maintenance of the pluripotency state is conferred by a range of development-associated transcription factors (such as OCT4, NANOG, SOX2) that reside in promoters of active genes associated with self-renewal [38]. The expression of these transcription regulators is typically controlled by CpG promoter methylation; SC differentiation is obtained by full or partial methylation of pluripotency-associated genes, leading to their downregulation [39]. Upregulation of these factors has been observed in the case of reprogramming of iPSCs from differentiated cells [38].

When it comes to epigenetic control of genes associated with self-renewal or differentiation, there are some dissimilarities between the SCs of embryonic and adult origin (Table 2). So, in ESCs, both Oct4 and Nanog genes are typically hypomethylated when activated and became hypermethylated during differentiation [40,41], whereas in MSCs of diverse origin, OCT4 is silenced by promoter hypermethylation, but Nanog and Sox2 are unmethylated despite the repressed state of the genes [42].

### 3.2. Histones Modification

Histones modification—such as acetylation/deacetylation—represents fundamental mechanisms that modulate gene expression by altering the chromatin structure. The balance between the acetylated/deacetylated states of histones is mediated by two sets of enzymes: histone acetyltransferases (HATs) and histone deacetylases (HDACs), having opposite effects on gene expression. Histone acetylation decreases the histone–DNA interactions, activating transcription, while deacetylation tightens histone–DNA interactions, leading to chromatin condensation and subsequent transcription inhibition [25].

Existing data advocate a critical role of HDAC in determining SCs’ fate. For example, it has been shown that over-expression of HDAC3 stimulated Sca-1^+^ cells to endothelial-lineage commitment [43]. Moreover, when exposed to laminar shear stress, differentiation of ESC-derived progenitor cells into functional endothelial cells has been promoted in an HDAC-dependent manner through activation of the Flk-1–PI3K–Akt pathway [44]. As for the influence of HDAC1 on the differentiation of various SC types, conflicting data have been reported. While some studies indicated a suppressive role of HDAC1 as regards differentiation into cardiomyocyte lineage [45,46,47,48], others stated that HDAC1 favors SC differentiation by down-regulation of pluripotency genes [49,50].

## 4. Transcriptomics

The transcriptome is represented by the total set of RNA transcripts (mRNAs, rRNAs, tRNAs, non-coding RNAs) produced in a cell. It reflects the genes that are actively expressed at a certain time; therefore, the abundance of a specific RNA transcript in a sample is a reflection of the corresponding gene expression level. Unlike the genome, the transcriptome can vary depending on external environmental conditions [51].

In this section, we provide a brief summary of the current knowledge on the SC transcriptome, with a specific focus on non-coding RNAs, emphasizing data with potential application for expediting cardiac regeneration.

A comprehensive paper on the transcriptome profiles of MSCs revealed source-specific markers. When comparing bone marrow-derived MSCs (BM-MSCs) with embryonic stem cell-derived MSCs (ESC-MSCs), 2500 differently expressed genes were identified. Specifically, 71 transcripts enriched in extracellular vesicle proteins were found exclusively in BM-MSC, endorsing once again the essential role of extracellular processes in MSC biology. As for the 19 ESC-MSC-specific transcripts, these include transcription factors or regulators involved in developmental processes (such as HOXD1, NKX2–5, LHX2 and FGF12), reflecting the superior differentiation potential [52]. It is of note that the rest of the ESC-MSC-specific transcripts are unknown, corresponding to neither genes nor noncoding RNAs.

Valuable information regarding endothelial progenitor cell (EPC) subtypes has been uncovered by the analysis of their transcriptome. It is widely acknowledged that the term “EPC” encompasses various subpopulations of progenitor cells, hence the conflicting results and misunderstanding concerning the role of these cells in health and disease [53]. The study conducted by Medina and colleagues provides a broad molecular fingerprint of two EPCs subtypes categorized according to the time at which they appear in culture: early EPCs and outgrowth endothelial cells (OECs), emphasizing once again the differences between them. As the authors stated, these cells have strikingly different gene expression signatures: early EPCs proved to be enriched in haematopoietic-specific transcripts (such as RUNX1, WAS, LYN), while OECs highly expressed transcripts involved in vascular development and angiogenesis (such as Tie2, eNOS, Ephrins) [54]. These data are of particular importance when in pursuit of possible candidates for prompting therapeutic angiogenesis; thus, OECs could be a suitable choice for cardiac protection and regeneration, as opposed to early EPCs.

A novel and promising method for directing the progenitor cell fate and homing and also for cellular reprogramming is based on in vitro-transcribed mRNAs. Compared to genome-integrating vectors, the main advantage of this method is that the mRNA molecules are not incorporated into the host genome, and therefore no mutations are triggered. These synthetic mRNAs can be used to prompt SC differentiation into a particular cell type, to promote the expression of receptors involved in SC migration and homing, or even to stimulate the production of desired human growth factors [55]. For example, intramyocardial injection of synthetic modified RNA encoding human vascular endothelial growth factor A (VEGF-A) markedly improved heart function and enhanced survival in a myocardial infarction model in mice [56].

### 4.1. MicroRNAs

MicroRNAs are small non-coding RNAs that regulate gene expression at the post-transcriptional level by translational repression and/or messenger RNA (mRNA) degradation, thus affecting a variety of cell processes.

There is a growing body of evidence supporting the key role of microRNAs in controlling SCs’ pluripotency, self-renewal and differentiation, as illustrated by detailed reviews [57,58,59,60].

Moreover, microRNAs are discussed as potential specific biomarkers that modulate various signaling pathways and cellular processes, and are involved in cell-to-cell communication both in physiological and pathological cardiovascular conditions [61,62].

As an example, miR-126 improves MSCs migration, survival and is also involved in angiogenic signaling in endothelial cells. Therefore, miR-126 overexpression in SCs increases angiogenesis and myocardial recovery in cardiac ischemia [63,64,65]. The implicated mechanism is related to the activation of the pro-survival Akt signaling pathway due to the suppression of PI3K inhibitors, resulting in enhanced cell survival and the amplified release of paracrine factors. On the contrary, mice deficient in miR-126 showed an impaired postnatal neovascularization after myocardial ischemia [66].

Other microRNAs involved in cell survival are miR-21, miR-24, and miR-221 which target the apoptotic protein Bim and consequently boost the survival and function of transplanted CSCs [67]. Furthermore, the overexpression of miR-210, miR-99, miR-21 and miR-214 reduces cell apoptosis and protects heart function after AMI. The protective function of these microRNAs is realized through positive regulation of angiogenesis and anti-apoptosis by targeting an anti-angiogenic factor that induces apoptosis (miR-210), by enhancing autophagy (miR-99) and by downregulating fibronectin and collagen expression to reduce fibrosis in infarcted heart (miR-21) [68]. Notably, conflicting evidence exists regarding the regulatory role of miR-21 on cardiac fibrosis. It has been showed that overexpression of miR-21 reduced collagen scar formation following AMI by decreasing the expressions of collagen I and fibronectin, and also by lowering the number of α-SMA-positive cells [69]. As opposed to the aforementioned finding, Gupta and colleagues demonstrated that miR-21 promoted monocyte to fibrocyte transition, while genetic and pharmacological inhibition of miR-21 successfully reduced fibrosis and fibrocyte accumulation in a murine model of heart transplantation [70]. Further studies are warranted in order to determine the exact roles of miR-21 in cardiovascular diseases in humans.

MicroRNAs also regulate SC differentiation. Intensification in cardiac lineage commitment has been reported after overexpression of various microRNAs (e.g., miR-1, miR-133, miR-208 and miR-499) in cultured or transplanted ESCs [61,71,72,73,74]. For example, miR-1 prompts differentiation of ESCs into cardiac phenotype by targeting HDAC4, resulting in activation of myocyte enhancer factor 2 (MEF2) transcription factor.

It is noteworthy that some of the microRNAs that control SC proliferation and differentiation are also associated with extracellular matrix turnover and therefore play a strategic role in cardiac regeneration in IHD. Such is the case of miR-1; a list of all 12 microRNAs with the aforesaid dual role is provided in the paper by Prathipati and colleagues [61].

Developmental studies using mouse models have revealed that a number of miRNAs are involved in cardiac regeneration by stimulating cardiomyocytes proliferation. For example, overexpression of miR-590 and miR-199a promotes cardiomyocyte proliferation both in neonatal and adult mice and has conserved long-term cardiac function after AMI [75].

MiRNAs have been used efficiently to epigenetically reprogram fibroblasts into cardiomyocytes. A mixture of four microRNAs (i.e., miR-499, miR-1, miR-133 and miR-208) has demonstrated the capacity to directly reprogram fibroblasts into cells that express cardiomyocyte-specific genes and proteins in vivo and in vitro [76]. The same effect has been observed using an alternative technique—delivery of lentivirus expressing the same miR cocktail into the injured heart, with significantly improved cardiac function and reduced infarct size [76].

### 4.2. Long Non-Coding RNAs

Long non-coding RNAs (lncRNAs) are transcripts greater than 200 nucleotides localized mainly in the nucleus, as opposed to mRNAs which are abundant in the cytoplasm. Although lncRNAs do not encode proteins, they have emerged as key regulators of gene expression, with critical functions in the proliferation and differentiation of SCs of embryonic and adult origin [68,77,78].

In recent years, a variety of cardiovascular-associated lncRNAs have been identified, both in humans, as well as in animals. The first one described was Braveheart (Bvht). By means of various strategies for ESC differentiation, it has been demonstrated that Bvht is indispensable for cardiovascular lineage commitment; in this respect, it works as an epigenetic modulator, activating a core of the cardiovascular gene network [79].

In their endeavor to decipher the transcriptome in human cardiac progenitor cell (CPC) differentiation, Ounzain and colleagues identified 570 lncRNAs that were modulated during cardiac commitment. As expected, many of these were associated with active cardiac enhancers or super enhancers. Among the studied lncRNAs, CARMEN (Cardiac Mesoderm Enhancer-Associated Noncoding RNA) is essential for cardiac specification and differentiation of human CPCs. This was evidenced by the fact that knockdown of CARMEN inhibits the differentiation of CPCs [80].

However, lncRNAs are also regulators of pluripotency. In a recent study, Loewer et al. identified a variety of lncRNAs whose expression is linked to pluripotency. Amongst these, 10 lncRNAs have been found to be upregulated in iPSCs, independent of the cell-of-origin, compared with ESCs, suggesting that their activation may promote reprogramming. Additional loss- and gain-of-function experiments confirmed the critical role of lncRNAs—such as LincRNA-Regulator of Reprogramming (LincRNA-RoR)—in the derivation of iPSCs [81].

Lnc RNAs may serve as potential therapeutic targets in the future, but there are a few downsides to their use, one of the most important being the fact that lncRNAs regulate a genetic network and not a single pathway (as summarized in Table 3); therefore, inhibition of lncRNAs might lead to severe, unforeseen complications [68].

## 5. Proteomics

### 5.1. Secreted Factors 

Proteomics, defined as the study of protein abundance and its variations at a certain time, can provide new valuable information about cell signaling mechanisms, considering the fact that proteins are the actual mediators of most cell processes [51,96]. Various extracellular factors, such as cytokine and matrix factors, influence SC self-renewal and differentiation via intracellular signaling pathways. More than one factor may be capable of triggering similar cell responses such as proliferation, differentiation, migration, and cell death [97]. Analyzing and comparing different SC proteomes can uncover vital biological processes related to SC behavior. Cutting-edge technologies of protein identification and quantification, along with tailor-made bioinformatics tools, enabled in-depth characterization of the SC proteome [98].

Billing and colleagues performed a comprehensive characterization of human MSCS, revealing source-specific cellular markers. By using high-resolution nano-liquid chromatography–mass spectrometry based on stable isotope labeling with amino acid in cell culture (SILAC) techniques, they conducted a thorough analysis of BM-MSCs, ESC-MSCs, and ESCs respectively [52]. While over 3000 proteins were differentially expressed between MSCs (regardless of origin) and ESCs, only 34 proteins were differentially expressed between BM-MSCs and ESC-MSCs. Furthermore, the authors compared their data to the most wide-ranging human proteome maps available, encompassing nearly 20,000 proteins of adult and fetal origin [99,100]. As expected, some of the up-regulated proteins in all three types of studied cells were related to transcription-related proteins. Bioinformatics analysis yielded similarities between the two types of MSCs, but also discrepancies; of note for our topic, as opposed to ESC-MSCs which displayed higher enrichment for proteins involved in neuron and axon development, BM-MSCs were more enriched for proteins regulating vasculature development [52].

Although in vitro systems are a very powerful tool capable of studying cell populations under controlled conditions, in vivo studies are the most reliable. One of the mechanisms by which SCs modulate the repair process is secretion of paracrine factors having various protective roles (e.g., angiogenic, mitogenic, anti-apoptotic, anti-inflammatory, and anti-oxidative) (Table 4).

The most intensely studied types of SC in terms of secretome are MSCs. Existing data advocate that these cells are capable—both in vitro and in vivo—of expressing and releasing powerful regulatory molecules such as VEGF, basic fibroblast growth factor (FGF-2), angiopoetin-1 (Ang-1), insulin-like growth factor-1 (IGF-I), hepatocyte growth factor (HGF), transforming growth factor β (TGF-β), interleukin-6 (IL-6) and stromal cell-derived factor-1 (SDF-1) [101,102,103,104]. Through these factors, MSCs not only promote the angiogenesis, but also support SC recruitment from bone marrow and improve the survival of resident cardiomyocytes. For instance, SDF-1α is an important chemoattractant for various progenitor cells with an essential role in endogenous SC migration and homing (through activation of CXCR4), adhesion, and recruitment of circulating MSCs and EPCs to the injured region of the myocardium. Furthermore, SDF-1 pretreatment can protect the resident cardiomyocytes from apoptosis, and improve their survival by paracrine secretion of FGF2 and VEGF [102,103].

As for the secretome of other SCs types, evidence suggests that EPCs also exhibit a high expression of angiogenic growth factors such as VEGF, SDF-1 and IGF-1 [105]. In a study conducted by Urbich and colleagues, it was demonstrated that conditioned media from EPCs prompted a robust migratory response of mature endothelial cells, and also of resident cardiac c-kit^+^ progenitor cells, supporting the assertion that the efficiency of EPC-induced cardiac regeneration may not only be determined by the incorporation of EPC into newly formed vessels, but may also be influenced by the release of secreted factors having synergistic effects [107].

Some of the findings discovered in preclinical studies have already been translated into clinical trials. Specifically, lineage specification by means of a cardiogenic growth factors cocktail was used by Bartunek and collegues to drive patient-derived BM-MSCs toward a cardiopoietic phenotype. Cardiopoietic SCT was found feasible and safe; moreover, compared to standard-of-care alone, reduction in left ventricular end-systolic volume, associated with improvements in 6-min walk distance and quality of life have been observed in patients with severe cardiac dilatation [108,109]. 

The impact of SDF-1 short-term overexpression on patients with ischemic HF has been addressed by STOP-HF trial (ClinicalTrials.gov Identifier NCT01643590). Although the composite primary end point was not achieved (improvement in 6-min walking distance and quality of life from baseline to 4 months), the investigators detected a significant improvement of left ventricular systolic function and a trend toward diminished negative remodeling in patients with more severe cardiac dysfunction [110].

### 5.2. Genetic Modification of SCs 

However, after many trials, the outcome of SCT is not as revolutionary as expected [111] and one of the problems seems to be the low survival rate of transplanted cells due to the hostile microenvironment of an ischemic heart. As a result, various strategies have been developed to enhance the survival and regenerative potential of transplanted cells [112,113,114]. Particularly, the administration of genetically modified SCs has emerged as a more advantageous method compared to direct gene transfer or therapy with non-modified SCs.

MSCs are the foremost type of SCs to be manipulated in this respect. For example, MSCs can be genetically modified to express VEGF in order to enhance their cardioprotective and angiogenetic properties when transplanted in acute or chronic ischemic settings [115,116].

Similarly, Akt-engineered MSCs have been demonstrated to improve cardiac function, by enhancing the left ventricular ejection fraction and diminishing scar size and fibrosis. The main mechanisms revealed to contribute to cardiac protection and functional improvement are better resistance to apoptosis and increased secretion of paracrine mediators such as VEGF, FGF-2, HGF, IGF-I, and thymosin beta-4 (TB4) [117,118,119]. Moreover, in a study conducted by Jiang and colleagues, it has been showed that the co-overexpression of Akt and angiopoietin-1 in transplanted MSCs led to better results in terms of cell survival, angiomyogenesis, and consequently improved cardiac function [120].

Another promising target for the genetic modification of SCs is the human tissue kallikrein (KLK1) gene with a proven protective role in cardiovascular disease both in vitro and in vivo [121]. KLK1 exerts significant cardioprotective effects by various underlying mechanisms, such as reducing myocardial inflammation and fibrosis, decreasing infarct size, increasing NO synthesis, restoring coronary blood flow, stopping cardiomyocyte apoptosis and promoting neo-vascularization [122,123,124,125,126,127]. Additionally, in a mouse model of myocardial infarction (MI), KLK1 gene delivery has been revealed to increase the number of resident CPCs and boost the regional blood flow and neo-vascularization in the peri-infarcted myocardium [128]. Therefore, genetically modified SCs seem to be a very appealing therapeutic option to deliver KLK1 to injured hearts. Indeed, genetic modification of MSCs with KLK1 led to superior neo-vascularization of the infarct myocardium due to increased VEGF secretion [125,129]. Moreover, KLK1-MSCs appear to be more resistant to hypoxia-induced apoptosis compared to MSCs, and they also seem to decrease myocardial apoptosis after MI by reducing capsase-3 activity [122].

Transfection of MSCs with genes encoding other pro-survival proteins or angiogenic factors has also been tested in preclinical studies, with encouraging results. Among the most exploited genes promoting the cell survival and function of infarcted myocardium are those coding for heme oxygenase-1 (HO-1), tumor necrosis factor receptor (TNFR), integrin-linked kinase (ILK), SDF-1, IGF, FGF and HGF [130,131,132,133,134,135,136].

However, MSCs are not the sole type of SC to have been genetically engineered in order to improve their regenerative potential. EPCs have also been modified in this respect. KLK1-transfected EPCs displayed enhanced functional capacity in vitro, as well as in vivo. When cultured, KLK1-EPCs exhibit superior abilities in terms of differentiation, migration, and vascular tube formation as compared to non-modified EPCs. More importantly, in vitro results have been supported by in vivo data. Subsequent assessments of functional capacity in a mouse model of AMI revealed significantly reduced cardiomyocyte apoptosis, increased retention of transplanted EPCs, and increased angiogenesis in the infarct border zone [137,138]. These beneficial effects were also observed after transplantation of EPCs genetically modified by the adeno-associated viral vector delivering the IGF-1 gene after myocardial infarction [139].

It has been widely acknowledged that the adult heart is endowed with a regenerative capacity due to the presence of the resident CSCs and their progenitors [140,141,142,143]. However, the endogenous system is not sufficient to efficiently repair the failing myocardium, especially since CSCs’ function further decreases with age and cardiovascular factors [144,145,146]. So, substantial efforts have been made to boost their regenerative potential. Indeed, engineering of human CSCs to overexpress IGF-1 improved cell-mediated healing by enhancing the long-term survival and engraftment of transplanted cells within the surrounding myocardium [147].

An additional appealing strategy to prime transplanted cells is to transfect them with the Pim1 gene. Pim1 is a highly conserved serine-threonine kinase which regulates cardiomyocyte survival downstream of Akt [148]. Studies conducted by Mohsin and colleagues revealed that Pim1-modified human CPCs display phenotypic characteristics consistent with increased survival, reversal of senescent characteristics, enhanced mitochondrial activity and cardiac commitment. Furthermore, when injected into a mouse model of AMI, Pim1-CPCs led to better cellular engraftment and differentiation with improved neovascularization and reduced infarct size [149,150].

## 6. Metabolomics

Integrating metabolomics (the study of the complete set of small molecules or metabolites in a cell) with the other omics approaches might provide a better understanding of the complex regulatory mechanisms of SCs [151]. The metabolite levels reflect metabolic function, with perturbation of these levels often being indicative of disease [51]. Therefore, metabolism, being directly or indirectly involved with every aspect of cell function, plays a crucial role in SC survival, differentiation and proliferation [151] (Table 5).

It has been demonstrated that bioengineered SCs undergo a conversion from oxidative metabolism to glycolysis, essential for their maintenance and self-renewal [157]. This suggests a potential role of glycolysis in SC self-renewal. On the other hand, during cardiogenesis, there is a switch from glycolysis to oxidative phosphorylation that drives ESC cardiac differentiation [96].

ESCs also rely on glycolytic energy generation [154,155], with pluripotency maintenance sustained under hypoxic conditions [152]. Nuclear reprogramming sets in motion dedifferentiation processes, leading to the acquisition of pluripotency. Mohyeldin and colleagues attested that hypoxia-mediated activation of glycolytic metabolism increased the efficiency of nuclear reprogramming, and maintained the pluripotent state [96,153].

By altering the epigenome, metabolites can control the fate of SCs [151]. A recent study showed that let-7 is the most highly up-regulated microRNA family during in vitro human cardiac maturation. Maturation of ESC-derived cardiomyocytes has been enhanced by the overexpression of let-7g and let-7i which promoted the metabolic switch to fatty acid oxidation [158].

Another epigenetic mechanism by which metabolites can control SCs’ fate is S-adenosyl methionine (SAM). SAM donates methyl groups for histone and DNA methylation; the levels of intracellular SAM can regulate methylation potential. Several metabolites, such as methionine and threonine, have been shown to affect SAM levels. Deprivation of methionine or threonine in culture medium (in mice) led to a rapid decrease in SAM, and triggered histone and DNA demethylation, thereby increasing SC differentiation. Conversely, SC culture in methionine-deprived medium resulted in increased apoptosis [151].

## 7. Exosomics

At the present time, the role of various extracellular vesicles in intercellular communication is widely acknowledged. The term “extracellular vesicles” denotes spherical membrane fragments originating from different subcellular compartments and generally discriminated by their size range. Explicitly, the terminology includes exosomes (40–150 nm diameter), microvesicles (100−1000 nm) and apoptotic bodies (1000−5000 nm). While exosomes are derived from the endosomal cell compartment by exocytosis, the larger vesicles are shed from the cell surface by budding and blebbing of the cell membrane [159,160]. Although all the abovementioned types of vesicles are carriers of omic information by harboring a wide array of macromolecules, we will focus only on exosomes since these have been studied the most as regards the potential to regenerate the heart.

Previous studies have shown that exosome cargo consists of proteins, lipids and, most importantly, various forms of RNAs that enable cell-to-cell communication and signaling [161,162]. What is more, under appropriate stimuli, exosomes migrate to specific tissues—such as ischemic myocardium—and deliver their cargo where needed [163]. As a result, in-depth analyses have been performed in the latest reviews exploring the strategic role of SC-derived exosomes in cardioprotection [164,165,166]. Existing data support the assertion that SC-derived exosomes (SC-Exos) exert similar biological effects, in terms of anti-apoptotic and pro-regenerative properties, to their releasing cells, endorsing them as surrogates of SCT. Indeed, in animal models of myocardial infarction, exosomes derived from MSCs had beneficial effects on blood flow recovery, infarct size, cardiac apoptosis and fibrosis, resulting in improved cardiac function [167,168,169]. The underlying mechanism through which MSC-Exos exercise their cardioprotective abilities seems to be activation of pro-survival signals—such as PI3K/Akt and Wnt/β-catenin pathways; moreover, in the hearts of MSC-Exos-treated animals, increased levels of ATP and NADH and decreased oxidative stress were detected [163,170].

MSC-Exos have also been used to prime other types of SCs. In vitro experiments evidenced an enhanced proliferation, migration, and angiogenic potency of CSCs preconditioned with MSC-Exo in a dose-dependent manner. In rats with myocardial infarction, injection of treated CSCs led to augmented engraftment and capillary density, reduced fibrosis, and notably a better cardiac outcome compared with CSCs only or the control [171].

Recently, the cardioprotective effect of SC-Exos has been tested in large animal models. In this respect, exosomes secreted by human cardiosphere-derived cells have been delivered in acute and chronic porcine myocardial infarction, either intracoronary or by the percutaneous intramyocardial route. Of note, solely exosomes with intramyocardial delivery reduced infarct size and preserved cardiac function [172].

In attempting to determine the mechanism by which SC-Exos stimulate cardiac regeneration, many researchers have focused on deciphering their content. Besides highly conserved proteins enclosed by most exosomes (such as tetraspanins and heat-shock proteins), there are also distinctive proteins that reveal their origin [173]. As mentioned in the “PROTEOMICS” section, SCs are able to secrete numerous cytokines, chemokines and growth factors which mediate their paracrine effects. So, in recent years, efforts have been made to decode the proteome of SC-Exos. Over 700 proteins have been identified in exosomes released by MSCs [174,175]. Among the proteins identified during proteomics analysis, LAMP2 and CD90 have been shown to be enriched in exosomes as compared with originating cells, while the metallopeptidase inhibitors TIMP-1 and TIMP-2 have been detected only in MSC-Exos [176]. Enrichment in the above-mentioned proteins explains the improved anti-remodeling potency of MSC-Exos.

One of the key constituents by which exosomes transfer functional information is represented by microRNAs. SC-Exos, enriched in specific miRNAs, promote cardiac regeneration by various mechanisms. When selecting the source of exosomes, one should keep in mind that exosomes are characterized by a distinctive miRNA signature, as are their parent cells. Therefore, ESCs have been considered an attractive source of exosomes aiming to stimulate endogenous progenitor cell proliferation and differentiation. Khan and colleagues reported significant enrichment of ESC-specific miRs—particularly that of the miR-290 family (namely miR-291, miR-294 and miR-295) in exosomes released by ESCs. Importantly, these miRs were further detected both in cultured CPCs and the hearts of animals treated with ESC-Exos. In vitro ESC-Exos administration led to increased survival and proliferation of CPCs, while in vivo data revealed improved cardiac function in treated animals compared to the control group [92].

However, not only have ESC-Exos been proven to have cardioprotective effects mediated by miRNAs, but also adult SC-Exos have been successfully tested in this regard. MiR-22 loaded exosomes secreted by MSCs-ameliorated fibrosis, reduced ischemia-induced apoptosis and improved cardiac function post-myocardial infarction [84].

The pro-angiogenic capacity of MSC-Exos was also investigated by in vitro as well as in vivo tests. After detailed quantification of 26 pro-angiomiRs in MSC-Exos, Gong et al. concluded that four of them (miR-30b, miR-30c, miR-424 and let-7f) were implicated in MSC-mediated angiogenesis. What is more, by using loss- and gain-of-function experiments, they demonstrated that miR-30b was important for in vitro stimulation of endothelial proliferation [85].

As for exosomes originating from cardiosphere-derived cardiac cells, studies have shown enrichment in several miRNAs (including miR-146a, miR-181b, and miR-126), with miR-146a and miR-181b being of particular interest for cardioprotection [86,87].

Similarly, exosomes derived from CD34^+^ cells are enriched in miR-126, known to have proangiogenic activity, therefore improving neovascularization after ischemia [177]. A low level of circulating miR 126 was observed in patients with diabetes or coronary artery disease, explaining the known impaired neovascularization capacity in these patients [178].

Additional data confirmed that SC-Exos miRNA secretome is modified in response to various pathological conditions, with either positive or negative outcome. Accordingly, exosomes generated by SCs grown under hypoxic conditions have reduced levels of miR-320 (shown to be anti-angiogenic), miR-222 (pro-apoptotic and anti-migration), and miR-185 (pro-fibrotic) as compared with exosomes produced by normoxic SCs. Additionally, a number of pro-angiogenic and anti-fibrotic miRNAs are upregulated in hypoxic exosomes, leading to diminished cardiac fibrosis and improved cardiac function. It also appears that exosomal miRNA content is regulated based on the length of time of parent SCs’ exposure to hypoxia [179,180].

As for complex cardiovascular comorbidities, metabolic syndrome has been proven to severely alter the miRNA cargo within SC-Exos. Of a total of 326 miRNAs identified in exosomes released by porcine adipose-derived MSC, eight were enriched in exosomes from animals having metabolic syndrome. Unsurprisingly, amongst upregulated miRNAs were those associated with decreased insulin sensitivity and ROS-induced tissue injury [181]. These findings are of a particular interest since most patients with IHD or HF have a number of comorbidities, and hence impaired function of SCs and SC-Exos. Therefore, even though current data endorse SC-Exos as promising candidates for cell-free therapy, effective strategies to boost their cardioprotective effect are required, especially in older patients with multiple comorbidities.

## 8. Challenges in Omics Data Management

First, one of the challenges faced when dealing with omics data is related to information retrieval, integration and analysis. The latest advances in the omics arena, as well as associated technologies, have yielded a huge amount of data which need to be wisely organized and rapidly disseminated to the scientific community. A large array of data is publicly available through various online database resources, so finding the right piece of information is not an easy task. There is a stringent need for developing dedicated informatics tools to assist researchers not only to analyze their own data, but to promptly retrieve information existing in the literature. In order to address this gap, in 2013 the first manually curated metadatabase was launched, encompassing over 4400 web-accessible tools related to genomics, transcriptomics, proteomics and metabolomics [182]. OMICtools was designed to cover all high-throughput technologies, and also to serve as a handy tool for bioinformaticians, researchers and clinicians.

However, when results of bioinformatics analyses need to be pinpointed in a findable, accessible, interoperable and reusable manner, one might use Datasets2Tools. Datasets2Tools is a repository comprising over 30,000 bioinformatics analyses, over 6000 biological datasets and over 4000 computational tools. This freely available platform was aimed at expediting the dissemination of digital resources and retrieval of information from biomedical research data [183].

Secondly, omics data should be seen as a whole, with each type of data being a piece of the puzzle which needs to be integrated with the other pieces in order to fully elucidate the intrinsic mechanisms underlying SC-mediated cardiac protection and regeneration [51,184,185].

What is more, specifically when facing heterogeneous SC populations, studies based on bulk tissue sampling do not accurately reflect biological processes carried out at the individual cellular level. Hence, integrated single-cell genome, epigenome, transcriptome and metabolome analysis is a more appropriate approach, but it does not come without challenges (i.e., the efficient isolation of individual cells and the low quantity of starting materials) [186].

## 9. Conclusions

Integrated omics (by systems biology) is a new innovative approach that could offer a better understanding of the intertwined cellular networks by creating a physiological and pathological cardiac blueprint. This might be a stepping stone to gaining the ability to regenerate the heart in vitro, and finally in vivo. Moreover, deciphering one’s ‘panomic’ data could trigger patient-tailored therapy, and change the face of cardiovascular medicine as we know it.

## Figures and Tables

**Table 1:** Genomic abnormalities in cultured human SCs and affected genes related to pluripotency, cell cycle, growth and apoptosis.

SC Type	Abnormality Type	% of Abnormal Cell Lines	Passage Number	Affected Gene	Encoded Protein	Protein Role	Ref.
ESCs, iPSCs	Trisomy 12	12–20	14	NANOG	Homeobox protein NANOG	Pluripotency	[7,8,9,10]
				GDF3	Growth differentiation factor-3	Pluripotency	
	Trisomy 8	9–20	19–26	PTP4A3	Protein tyrosine phosphatase type IVA, member 3	Cell proliferation	[7,8,11]
				NDRG1	N-myc downstream regulated 1	Cell growth	
	Trisomy X	1–5	5–8	FAM58A	Family with sequence similarity 58, member A	Cell proliferation	[7,8,11]
	CNVs (20q11.21)	24–80	24–76	ID1	Inhibitor of DNA binding 1	Cell growth	[7,11,12,13,14]
				BCL2L1	B-cell lymphoma-extra large	Anti-apoptotic	
				PDRG1	p53 and DNA damage-regulated protein 1	Cell survival	
				TPX2	Targeting protein for Xklp2	Cell cycle	
				KIF3B	Kinesin Family Member 3B	Cell cycle	
MSCs	Trisomy 8	4	7	PTP4A3	Protein tyrosine phosphatase type IVA, member 3	Cell proliferation	[10,15]
CSCs	Trisomy 8 (normoxia vs hypoxia)	31 vs 0	1 vs 6	PTP4A3	Protein tyrosine phosphatase type IVA, member 3	Cell proliferation	[16,17]
ADSCs	Trisomy 8	8–12	2	PTP4A3	Protein tyrosine phosphatase type IVA, member 3	Cell proliferation	[18]

Ref. = references.

**Table 2:** DNA-methylation of transcription factors associated with pluripotency in embryonic stem cells (ESCs) and mesenchymal stem cells (MSCs).

SC	Transcription Factor	Active State	Repressed State	Ref.
ESCs	OCT4	Hypomethylated	Hypermethylated	[40,41]
	NANOG	Hypomethylated	Hypermethylated	
MSCs	OCT4		Hypermethylated	[42]
	NANOG		Hypomethylated	
	SOX4		Hypomethylated	

OCT4 = octamer-binding transcription factor 4; NANOG = homeobox protein Nanog; Ref. = references; SOX4 = SRY-Box 4.

**Table 3:** Main non-coding RNAs expressed by SCs that regulate SC fate and cardioprotection.

Transcript	Source	Effect	Target Molecule/Pathway	Ref.
miR-21	MSC-Exos	↓ apoptosis	↓ inhibitors of pro-survival PI3K/Akt pathway	[60,67,82,83]
	CSCs, CSC-Exos	↓ apoptosis	↓ CASP3, CASP8AP2, BAX, PDCD4, FASL, BCL2L11, FOXO3, AK2	
		↑ proliferation and migration of CSCs	↓ PTEN, PDCD4	
	iPSC-Exos	↓ apoptosis	↓ CASP3/7	
miR-22	MSCs, MSC-Exos	↓ apoptosis; reduces cardiac fibrosis	↓ MECP2	[60,84]
	CSCs	↑ commitment to SMCs	↓ EVI1	
miR-24	CPCs	↓ apoptosis	↓ CASP3, CASP8AP2, BAX, PDCD4, FASL, BCL2L11, FOXO3, AK2	[67]
miR-30b	MSC-Exos	↑ angiogenesis	↓ endothelial Dll4	[85]
miR-30c	MSCs-Exos	↑ angiogenesis	↓ endothelial Dll4	[85]
miR-126	MSCs, EPCs EPC-Exos	↑ migration and survival of MSCs and EPCs; ↑ angiogenesis	↓ inhibitors of pro-survival PI3K/Akt pathway; ↑ Dll4 expression	[57,64,65,66,86]
	CDCs, CDC-Exos	↑ cardioprotection	↓ PKCδ expression	
miR-146a	CDC-Exos CPC-Exos	↑ cardioprotection, ↓ fibrosis	↓ IRAK1 and TRAF6	[87,88]
miR-181b	CDC-Exos	↑ cardioprotection	↓ PKCδ and MAP4K4	[86]
miR-199a	CSCs	↑ cardiomyocyte proliferation; ↓ apoptosis	↓ P53 activity	[75,89]
miR-208	MSCs	↑cell proliferation and clonogenicity	↓ AIMP3/p18 and senescence markers	[90]
miR-210	MSCs	↓ apoptosis	↓ CASP8AP2	[68,91]
	CDCs, CDC-Exos	↑ angiogenesis	↓ EFNA3	
	iPSCs-Exos	↓ apoptosis	↓ CASP3/7	
miR-221	CPCs	↓ apoptosis	↓ CASP3, CASP8AP2, BAX, PDCD4, FASL, BCL2L11, FOXO3, AK2	[67]
miR-291	ESC-Exos	↑ CPC survival and proliferation	↓ P53 activity	[92]
miR-294	ESC-Exos	↑ CPC survival and proliferation	↓ P53 activity	[92]
miR-295	ESC-Exos	↑ CPC survival and proliferation	↓ P53 activity	[92]
let-7f	MSC-Exos	↑ angiogenesis	↓ endothelial THBS1	[85,93]
Braveheart	ESCs MSCs	↑ commitment toward the cardiovascular lineage	Activates MESP1, GATA4, HAND1, HAND2, NKX2.5, TBX5, SNAI, TWIST	[79,94]
		↑ epigenetic activation of cardiac genes	Binds SUZ12	
CARMEN	CPCs	↑ cardiac specification and differentiation of CPCs	Interacts with chromatin remodeling complexes (PRC2)	[80]
LincRNA-RoR	iPSCs	↑ reprogramming	Suppresses P53 pathways	[81,95]
	ESCs	↑ self-renewal of human ESCs	Captures miRNAs targeting OCT4, SOX2, NANOG	

↓ = suppresses; ↑ = increases; AIMP3/p18 = eukaryotic translation elongation factor 1 epsilon 1; AK2 = Adenylate kinase 2; BAX = BCL2 associated X; BCL2L11 = Bcl-2 protein family; CASP3 = caspase 3; CASP8AP2 = CASP8-associated protein 2; Dll4 = Notch ligand Delta-like-4; EFNA3 = endothelial Ephrin-A3; EVI1 = ecotropic viral integration site 1; FASL = Fas ligand; FOXO3 = forkhead box O3; HAND1/2 = heart and neural crest derivatives-expressed protein 1/2; IRAK1 = interleukin-1 receptor-associated kinase 1; MAP4K4 = Mitogen-activated protein kinase kinase kinase kinase 4; MECP2 = methyl CpG binding protein 2; MESP1 = mesoderm posterior 1; NANOG = homeobox protein Nanog; NKX2.5 = NK2 homeobox 5; OCT4 = octamer-binding transcription factor 4; P53 = tumor protein; PI3K/Akt = phosphatidylinositol 3-kinase/ protein kinase B; PDCD4 = programmed cell death; PKCδ = protein kinase C δ; PRC2 = polycomb repressive complex 2; PTEN = phosphatase and tensin homolog; Ref. = references; SMCs = smooth muscle cells; SNAI = snail; SOX4 = SRY-Box 4; SUZ12 = SUZ12; TBX5 = T-Box 5; THBS1 = thrombospondin 1; TRAF6 = tumor necrosis factor receptor associated factor 6; TWIST = twist-related protein 1.

**Table 4:** Effects and signaling pathways of SC secreted factors mediating cardiac repair.

Factor	Source	Effect	Signaling Pathway	Ref.
FGF-2	MSCs	↑ MSC proliferation; ↑ angiogenesis; ↓ apoptosis	ERK1/2, PI3K-Akt pathways	[101,102,103]
TGF-β	MSCs	↑ MSC proliferation; angiogenesis; ↓ apoptosis	SMAD, PI3K/Akt, MAPK pathways	[101,102,103]
VEGF	MSCs, EPCs	↑ angiogenesis; ↓ apoptosis	PI3K/Akt, MAPK pathways	[101,102,103,104,105,106]
HGF	MSCs, CSCs	↑ angiogenesis; ↓ apoptosis	ERK1/2, p38 MAPKs, PI3K/Akt, NOTCH pathways	[101,102,103]
IGF-1	MSCs, EPCs, CSCs	↑ angiogenesis; ↓ apoptosis	ERK1/2, PI3K-Akt pathways	[101,102,103,105]
Ang-1	MSCs	↑ angiogenesis	Tie-2 pathway	[101,102,103]
SDF-1	MSCs, EPCs	↑ mobilization and homing of BM-MSCs and EPCs; ↑ angiogenesis; ↑ migration and differentiation of CSCs; ↓ apoptosis	ERK1/2, PI3K-Akt pathways	[101,102,103,105]
IL-6	MSCs	↑ MSC proliferation and “stemness”; ↑ endothelial differentiation of CSCs; ↑ angiogenesis	ERK1/2, JAK-STAT pathway	[101,102,103]

↓ = reduces; ↑ = increases; Ang-1 = angiopoietin-1; ERK1/2 = extracellular signal-regulated kinase 1/2; FGF = fibroblast growth factor; HGF = hepatocyte growth factor; IGF = insulin growth factor-1; IL-6 = interleukin-6; JAK-STAT = Janus kinase/signal transducer and activator of transcription; MAPK = mitogen-activated protein kinase; PI3K/Akt = phosphatidylinositol 3-kinase/ protein kinase B; Ref. = references; SDF-1 = stem cell-derived factor-1; Tie-2 = tyrosine-protein kinase receptor; TGF-β = transforming growth factor β, VEGF = vascular endothelial growth factor.

**Table 5:** Regulation of SC fate by metabolites/ metabolic pathways.

Metabolite/ Metabolic Pathway	Effect	Ref.
SAM	Promotes pluripotency of ESCs and iPSCs	[151]
Hypoxia	Promotes pluripotency of ESCs and iPSCs; Promotes undifferentiated state of MSCs and expression of anti-apoptotic and angiogenic factors	[96,152,153]
Glycolysis	Promotes pluripotency of ESCs and iPSCs Promotes undifferentiated state of MSCs	[96,154,155,156]
Oxidative phosphorylation	Promotes cardiac differentiation of ESCs Promotes differentiation of MSCs	[96,156]

Ref. = references; SAM = S-adenosyl methionine.
